# Psychometric Validation of the Dating Violence Questionnaire (DVQ-R) in Ecuadorians

**DOI:** 10.3390/bs15010068

**Published:** 2025-01-15

**Authors:** Miriam Jacqueline Muñoz-Aucapiña, Rosa Elvira Muñoz-Aucapiña, Inmaculada García-García, María Adelaida Álvarez-Serrano, Ana María Antolí-Jover, Encarnación Martínez-García

**Affiliations:** 1 Department of Nursing, Faculty of Health Sciences, University Católica Santiago de Guayaquil, Guayaquil 090615, Ecuador; miriam.munoz@cu.ucsg.edu.ec (M.J.M.-A.); rosa.munoz@cu.ucsg.edu.ec (R.E.M.-A.); 2 Department of Nursing, Faculty of Health Sciences, University of Granada, 18016 Granada, Spain; igarcia@ugr.es (I.G.-G.); emartinez@ugr.es (E.M.-G.); 3 Department of Nursing, Faculty of Health Sciences, University of Granada, 51001 Ceuta, Spain; antolijover@ugr.es; 4 Virgen de las Nieves University Hospital, 18014 Granada, Spain; 5 Instituto de Investigación Biosanitaria ibs.GRANADA, Granada, Spain

**Keywords:** dating violence, nursing students, gender violence, validation study

## Abstract

Gender-based violence among young people is a pressing global problem, causing injury and disability to women and posing physical, mental, sexual, and reproductive health risks. This study aimed to psychometrically validate the Dating Violence Questionnaire—Revised (DVQ-R) in a sample of 340 Ecuadorian university students. The study included 340 male and female students from two universities in Ecuador. The reliability and validity of the questionnaire were rigorously assessed by exploratory and confirmatory factor analyses, which revealed a four-factor model as the most parsimonious solution (RMSEA = 0.012). The factors were labelled as follows: ‘emotional neglect and contempt’, ‘physical violence and aggression’, ‘coercion and control’, and ‘emotional manipulation and testing’. The validated scale yielded a Cronbach’s alpha (α) of 0.839, with individual alpha values of 0.872, 0.764, 0.849, and 0.729 for each dimension. Convergent validity was established, as the mean variance extracted per factor exceeded 0.4. Divergent validity was confirmed, as the variance retained by each factor was greater than the variance shared between them (mean variance extracted per factor > ϕ^2^). These results indicate that the DVQ-R is a valid and reliable instrument to assess dating violence among Spanish-speaking young adults, which supports future research and prevention programmes.

## 1. Introduction

Dating violence among young people is now reported in many countries (Gebrie et al., 2022; Oyarzún et al., 2021; World Health Organizatión, 2021). Its definition encompasses any type of physical violence, sexual violence, stalking, or psychological aggression (including coercive acts) by a current or former partner, both in adolescence and adulthood (Miller & McCaw, 2019).

Globally, dating violence has received increasing attention due to its high prevalence. In the United States, for example, according to a report by the Centers for Disease Control and Prevention (CDC), approximately 10% of college students report having experienced physical violence in their dating relationships, while 11% have experienced sexual violence in these relationships (Wiklund et al., 2010).

In Europe, statistics also reveal a high prevalence of dating violence. According to data from the European Union Agency for Fundamental Rights (FRA), 33% of young women have experienced some form of physical or sexual violence in their youth. This problem is recurrently observed in the university environment (Neilson et al., 2023), where according to studies carried out in 2015 by the American Association of Universities on 27 university campuses, more than 20% of university students report having been victims of sexual aggression (Association of American Universities, 2015). In Spain, the first research on violence against women in the university context revealed that 62% of students knew of or had experienced situations of this type within these institutions (Valls et al., 2016). In addition, cultural contexts seem to have a strong influence on students’ perceptions of IPV. In this context, studies suggest that university students in North America, predominantly in the United States, have less favourable attitudes towards IPV than their counterparts in Asia, South America, or Europe (Buquet Corleto, 2011; Zark & Satyen, 2022). On the other hand, An et al. (2023), in their study of Hispanic/Latino and non-Hispanic white students across seven universities, found that Hispanic/Latino students had higher rates of intimate partner violence victimisation and perpetration than their white counterparts. In a similar vein, an acceptance of violence in these populations, influenced by social norms, was found in Quito, Ecuador (Medina Maldonado et al., 2022).

Consequently, several studies have found that dating violence is a common experience among young adults, with prevalence differing across countries and contexts (Coulter et al., 2017). The existence of gender inequalities, patriarchal stereotypes, and socio-cultural norms that perpetuate violence play a key role in its persistence.

In Latin America, where strong gender disparities and very traditional behaviours prevail, dating violence is a serious problem (Red de Desarrollo Social de América Latina y el Caribe, 2017). In university environments, young people explore their dating relationships more freely (Kaukinen, 2014), and while some adopt communication skills to express their feelings, others adopt risky behaviours and various methods of physical, sexual, and psychological coercion to validate their love and affection in their dating relationships. It is significantly more common for women than men to be the victim in these situations (Bhochhibhoya et al., 2021).

In Ecuador, the situation is no different. The National Survey on Family Relations and Gender Violence published in 2019 indicates that six out of ten women between 15 and 45 years of age have suffered some type of violence at least once in their lives. The most frequent is psychological violence (1/2), followed by physical (2/5), patrimonial (4/10), and sexual (1/4). Of these women, 45.0% were between 15 and 17 years old (INEC, 2019).

However, there are no specific official data that provide a detailed analysis of the prevalence of dating violence among young people, which has limited the implementation of specific programmes to address and prevent this problem.

Considering that dating relationships in young people constitute a key aspect for the shaping of future interaction patterns in couple relationships (Gómez et al., 2014; Wiklund et al., 2010), their quantification represents a priority to identify the problem and promote measures aimed at the prevention and eradication of violence (Galdo Castiñeiras et al., 2023). To this end, different instruments have been developed, including self-administered questionnaires such as Aggression in Dating Situations (AADS), the Acceptance of Violence Questionnaire (AVQ), the Conflict Tactics Scale (CTS), the Conflict in Adolescent Dating Relationships Inventory (CADRI), and the Justification of Verbal/Coercive Tactics Scale (JVCT) (Yanez Peñúñuri et al., 2019). However, the Dating Violence Questionnaire (DVQ-R) is especially recommended for young people aged 15–26 years, due to its greater structural stability (Rodríguez Díaz et al., 2017). This instrument was created in Spain by authors such as (Rodríguez Franco et al., 2010), with a first version of 42 items, and has been widely used in research contexts to better understand the prevalence and characteristics of dating violence. It was later revised and modified, resulting in the DVQ-R version with 20 items. This modification, made to improve the reliability and validity of the scale, sought to simplify certain items and adapt them to reduce both cultural and interpretation biases that were observed in different contexts (Alfaro Urquiola, 2020). The revised version used in this study offers a more robust measurement and has been validated and used in different university settings in countries such as Spain, Mexico, Colombia, Peru, Argentina, and Bolivia, but so far it has not been validated in men and women in the Ecuadorian context (Alfaro Urquiola, 2020; Martínez Gómez et al., 2021; Rodríguez Díaz et al., 2017).

For all these reasons, universities have become ideal environments for exploring multiple aspects related to IPV in young people. The aim of this study was to carry out the psychometric validation of the Dating Violence Questionnaire (DVQ-R) in an Ecuadorian university population.

## 2. Materials and Methods

### 2.1. Design

In order to adapt and evaluate the psychometric properties of the DVQ-R, a cross-sectional study was conducted. The questionnaire was administered to young men and women from two Ecuadorian universities: the private Universidad Católica Santiago de Guayaquil and the public Universidad de Guayaquil. Data collection took place during the months of June to September of the academic year 2021/2022.

### 2.2. Sample, Participants, and Measures

The accessible population of the study was nursing students of various academic levels, corresponding to a total of 1657 students enrolled in the period 2021/2022.

Although in the process of validating a questionnaire, there is no single criterion for establishing the sample size, one of the most considered guidelines suggests having between 5 and 10 participants per item (The R Foundation, n.d.). Therefore, following this criterion, in the specific case of the DVQ-R composed of 20 items, a minimum total sample size of between 100 and 200 participants is suggested.

### 2.3. Instrument

The questionnaire was divided into two blocks. The first collected information on socio-demographic variables: year of birth; sex (male, female); university (Universidad Católica de Santiago de Guayaquil, Universidad Estatal de Guayaquil); relationship status (No, Yes); relationship duration (1–3 months, 3–6 months, 1–3 years, >3 years). The second block was based on the Dating Violence Questionnaire—Revised (DVQ-R) (Rodríguez Díaz et al., 2017), which was later validated for samples of Ecuadorian women aged 18 to 30 by Cherrez Santos et al. (2022). This study remains the ideal reference for exclusively female samples in this age group and population. This instrument is a simplified version of the Dating Violence Questionnaire (DVQ) (Rodríguez Franco et al., 2010). It is aimed at adolescents involved in dating relationships, or who have been in a relationship in the last six months, with a minimum duration of one month. It includes 20 items and uses a Likert-type scale with five response options (0 = Never to 4 = Always). It can be administered individually or in a group, lasting approximately five to ten minutes. This questionnaire assesses five dimensions of dating violence, which are shown below:

1. Coercive violence: explicit behaviours aimed at pressuring the partner to force his or her will or behaviour, represented by items 1, 9, 25, and 38.
2. Sexual violence: sexist or sexual behaviours, such as unwanted play on the part of the partner or feeling forced to perform acts and touching, represented by items 2, 10, 26, and 39.
3. Physical violence: includes hitting, shoving, direct injury, or indirect injury through damage to objects with emotional significance for the victim, represented by items 5, 13, 20, and 21.
4. Detachment violence: behaviours related to an attitude of indifference and discourtesy towards the partner and his or her feelings, represented by items 6, 14, 30, and 32.
5. Humiliation violence: behaviours of personal criticism directed against the partner’s self-esteem and personal pride, neglect, and denial of support, as well as behaviours aimed at lowering the esteem of the person, represented by items 15, 23, 40, and 41.

### 2.4. Validation of the Instrument

The content validity, internal consistency, construct validity, convergent validity, and discriminative validity of the DVQ-R were evaluated for the Ecuadorian youth population. In the adaptation process, a group of experts made up of nursing professionals with experience in both clinical and teaching settings, and who were familiar with the work of the DVQ-R, considered the discrepancies found by other authors and issued their opinion regarding the coherence, relevance, and thematic clarity of the instrument (Amar & Laughon, 2020). In addition, content validity was assessed by a group of 10 students with socio-demographic characteristics similar to those of the final sample.

Construct validity was assessed by exploratory factor analysis (EFA) and confirmatory factor analysis (CFA) in two stages. Exploratory factor analysis (EFA) was conducted to identify the dimensions of the questionnaire, followed by confirmatory factor analysis (CFA) to validate the factor structure, using the total sample.

EFA was performed using the principal component analysis (PCA) method with oblimin rotation. Each factor of the questionnaire was modelled as a variable and the number of factors was determined for eigenvalues greater than 1. To estimate the reliability of the questionnaire, the internal consistency of each factor was measured by calculating Cronbach’s alpha coefficient. Convergent validity was also calculated to estimate the average variance extracted (AVE). An AVE greater than 0.4 indicates that the measurement questions can better reflect the characteristics of each variable in the model (Owens et al., 2024). The discriminative reliability of the scale was tested using the correlation matrix between the factors.

To determine the overall fit of the proposed model, a CFA with the WLSMV (weighted least squares mean and variance adjusted) estimator was performed on the covariance matrix. In addition, a parallel CFA with the 5-dimensional structure described by Rodríguez Franco et al. (2010) was performed to compare the results. The WLSMV estimator was used to estimate the goodness-of-fit parameters, using additional indices to the C and additive fit indices (Knaul et al., 2020) mentioned by Bentler PM and Lévy Mangin (Cerdán Torregrosa et al., 2023; Dion et al., 2023; Romero Méndez et al., 2021).

### 2.5. Data Analysis

Descriptive analyses and AFE were performed using IBM-SPSS version 25 for MAC. On the other hand, CFA and figure analyses were performed with R version 4.4.1 (14 June 2024) in the R statistical environment using specific packages (The R Foundation, n.d.).

### 2.6. Ethical Considerations

The study was approved by the Ethics Committee of Hospital Clínica Kennedy HCK-CEISH-20-0031, Guayas province (Ecuador). All participants received a written information sheet and signed an informed consent form. In addition, the author of the original scale was contacted to request permission for the adaptation and validation of the questionnaire.

## 3. Results

### 3.1. Description of the Study Sample

The sample was used in its entirety for both exploratory factor analysis (EFA) and confirmatory factor analysis (CFA), which allowed us to explore and thoroughly validate the dimensions of the questionnaire. This approach ensures an adequate representation of the population studied, whose socio-demographic and relational characteristics are shown in Table 1.

### 3.2. Content Validity

An expert team of nurses with clinical and teaching experience who were familiar with the work of the DVQ-R confirmed that all proposed items were clearly worded, relevant, and consistent with the construct being measured, i.e., different forms of intimate partner violence victimization. To further enhance the validity of the instrument, content validity was also assessed by a group of 10 students whose socio-demographic characteristics closely matched those of the final sample. The feedback from this group helped ensure that the questions were understandable and relevant to the target population, reflecting their experiences and perceptions of dating violence. For those items where potential confusion was detected, additional explanations were included in parentheses to ensure greater clarity and facilitate respondents’ understanding.

### 3.3. Construct, Convergent, and Discriminant Validity

#### 3.3.1. Exploratory Factor Analysis (EFA)

In order to explore the best factor structure, an exploratory factor analysis (EFA) was conducted. The KMO test value was 0.895 and Bartlett’s test of sphericity was significant (*p* = 0.001). During this first analysis, the existence of four factors was demonstrated; although item 4 showed a communality lower than 0.5 (0.366), it was decided to keep it due to its valuable contribution to the construct.

In the final solution, eigenvalues greater than 1 showed the existence of four factors. This solution converged in five iterations and explained 59.73%. The items had factor loadings above 0.50 within their factor and communalities above 0.50 (Table 2). The factor loadings of all items were above the 0.50 threshold (0.537 to 0.811), and the average variance extracted (AVE) was above 0.4.

The Dating Violence Questionnaire presents four factors in the context of Ecuadorian culture with 20 items. The four factors identified were qualitatively labelled by the research team as follows: Factor 1 includes eight items related to the ‘Emotional Neglect and Contempt Dimension’: items 6, 14, 21, 26, 30, 39, 40, and 41. Factor 2 contains four items related to the ‘Physical Violence and Aggression Dimension’: items 5, 13, 15, and 20. Factor 3 contains four items related to the ‘Coercion and Control Dimension’: Items 2, 10, 25, and 38. Finally, Factor 4 contains four items related to the ‘Manipulation and Emotional Testing Dimension’: Items 1, 9, 23, and 32.

#### 3.3.2. Convergent and Discriminant Validity

Convergent validity was established, as the mean variance extracted per factor exceeded 0.4, with values of 0.48, 0.47, 0.60, and 0.40, respectively.

Discriminant validity between factors was confirmed when the variance retained by each factor was greater than the variance shared between them (AVE > ϕ^2^). The results demonstrated validity among the four factors, as detailed in Table 3.

#### 3.3.3. Internal Consistency

The internal consistency analysis is shown in Table 4. Cronbach’s alpha coefficient (α) was 0.839 for the total scale, and all factors scored above 0.7.

#### 3.3.4. Confirmatory Factor Analysis (CFA)

A CFA was then carried out to test the exploratory factor structure of the four-factor model. The results were compared with the five-factor model developed by Rodríguez Franco et al. (2010).

**Figure 1:**
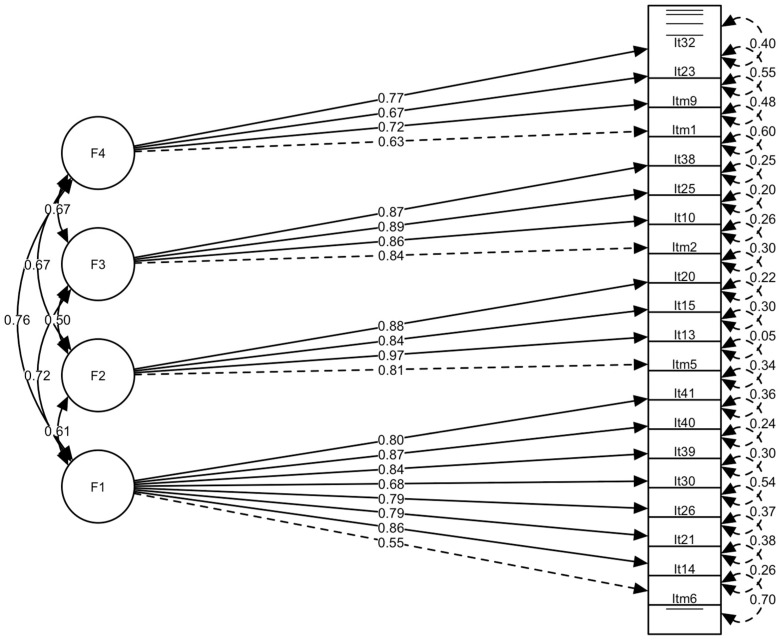
Confirmatory factor analysis of the four-factor model. Confirmatory factor analysis of the four-factor model, with standardised weights and measurement errors for each of the items included in the DVQ-R scale.

For the estimation of the goodness-of-fit parameters, the WLSMV estimator was used; the fit indices are presented in Table 5. Figure 1 shows the model with the normalized scores. The chi-square (χ^2^) values are statistically significant in both models; however, the four-factor measurement model presents a better fit, indicating that the items correctly reflected the latent constructs (Table 5). In particular, the RMSEA value of 0.012 indicates an excellent fit, well below the 0.05 threshold. In addition, the CFI and TLI values of 0.999 reflect a superior fit, exceeding the recommended threshold of 0.90. The GFI and AGFI values also support this conclusion, with values of 0.993 and 0.989, respectively.

### 3.4. Item Analysis

Table 6 shows a summary of the scores obtained for each factor of the questionnaire, providing a concise overview of the different types of violence experienced by the sample. In particular, Factor 4, ‘manipulation and emotional testing’, stands out with a mean of 2.03 (S.D. = 0.788). Next, Factor 1, ‘emotional neglect and contempt’, has a mean of 1.91 (S.D. = 0.806). Factor 3, ‘coercion and control’, has a mean score of 1.51 with a standard deviation of 0.403. Finally, Factor 2, ‘physical violence and aggression’, has the lowest score, with a mean of 1.23 (standard deviation of 0.235).

## 4. Discussion

To make progress in the fight for gender equality and the eradication of gender-based violence, it is crucial to address the existence of this problem (Amar & Laughon, 2020; Knaul et al., 2020; Owens et al., 2024; Verbeek et al., 2023). Moreover, authors such as Cerdan et al. and other researchers show that violence is currently occurring at increasingly earlier stages (Cerdán Torregrosa et al., 2023; Dion et al., 2023; Romero Méndez et al., 2021), which requires adequate identification and quantification. This study has attempted to evaluate the reliability, validity, and internal consistency of the Dating Violence Questionnaire—Revised (DVQ-R), adapted to the specific culture and context of Ecuador in a university population of men and women (Appendix A). It is therefore the first in this setting, thus joining those carried out in this line in other countries (Alfaro Urquiola, 2020; Martínez Gómez et al., 2021; Rodríguez Díaz et al., 2017). Furthermore, the results suggest that the questionnaire could be useful for research in other socio-cultural contexts in Latin America, given the similarity of cultural and social patterns in the region.

Compared to previous psychometric validation studies, substantial discrepancies have been observed in both the structure of the questionnaire and the characteristics of the sample. The original model of the DVQ-R postulated five dimensions, including sexual violence, whereas in our study, sexual violence did not emerge as an independent dimension, as Romero et al. have previously documented in Mexico (Anitha & Lewis, 2018). In fact, the prevalence of psychological dating violence shows higher incidence rates compared to physical and sexual violence. However, prevalence studies in high-income countries, such as the United States, Canada, the United Kingdom, and Australia, report levels of IPV that are mainly sexual (Terrazas Carrillo et al., 2024). Furthermore, the absence of sexual violence as a separate dimension might indicate that, in our sample, experiences of sexual violence are more closely related to other forms of maltreatment. This result underscores the interconnectedness of different types of violence in Ecuadorian university dating and highlights the importance of prevention strategies that address these interrelated complexities.

Also, a fusion of elements related to coercion and control was observed in the new dimension ‘coercion and control’, suggesting that experiences of violence may manifest differently in university contexts. This reinforces the importance of adapting measurement tools to the specific characteristics of the population studied (Campbell et al., 2021; Łukaszek, 2022; Mulumeoderhwa & Harris, 2015). The identification of the dimension ‘coercion and control’ as an independent factor highlights the uniqueness of these aspects among youth populations. The structural difference found here underlines the need to specifically address power and control dynamics in prevention interventions and policies, recognizing their importance in young adult relationships (De Sousa et al., 2023; Swan et al., 2021; Wong et al., 2023).

Furthermore, these discrepancies could be related to differences in the samples studied. Although young adults share some psychosocial traits with adolescents, they face different emotional and decision-making challenges, which may affect how they perceive and report dating violence (Łukaszek, 2022; Swan et al., 2021). This justifies the need to adapt and validate tools such as the DVQ-R for university populations, ensuring that they capture the specific dynamics of this age group.

Additionally, the separation of ‘manipulation and emotional testing’ as a distinct dimension reflects the complexity of emotional interactions in dating (Cherrez Santos et al., 2022). This suggests the need to address manipulation tactics and emotional testing separately when addressing dating violence.

To ensure the robustness of the revised structure of the DVQ-R, both internal consistency and construct validity were assessed. Internal consistency, as measured by Cronbach’s alpha, demonstrated high reliability for all four factors, ranging from 0.729 to 0.872. This strong consistency reflects the findings of other studies, reaffirming the reliability of the scale. Convergent validity was established, with an average variance extracted (AVE) greater than 0.4 for all factors, confirming that the items of each factor adequately capture the intended constructs. Discriminant validity was also achieved, as the variance retained by each factor was greater than the variance shared between factors (AVE > ϕ^2^). These results confirm that the four dimensions represent distinct and independent aspects of dating violence.

The revised four-factor structure was further supported by the model fit indices, which showed significant improvements compared to the original model. Specifically, the discrepancy between chi-square and degrees of freedom (CMIN/DF) improved from 3.849 in the original model to 1.052 in the revised model, while the root mean square error of approximation (RMSEA) decreased from 0.092 to 0.012, indicating an overall better fit. Other indices, such as the goodness-of-fit index (GFI) and the comparative fit index (CFI), also showed improvements, reaching near-perfect values in the revised model (GFI = 0.993, CFI = 0.999), confirming that the updated structure better represents the underlying data for this population.

Finally, the inclusion of both men and women in this study, as opposed to only women, as in Cherrez Santos et al. (2022), was intended to diversify the experiences captured in the DVQ-R. Our results are consistent with those documented by Sanmartín-Andújar et al. (2023), in which the largest differences between men and women were observed in the items of the control domain. This is consistent with multiple studies suggesting that relationship dynamics vary significantly between sexes. These differences in gender dynamics suggest that interventions and prevention strategies should be tailored to address the unique experiences of men and women, especially in terms of power and control (Vives Cases et al., 2021).

In addition, this study represents a first effort to validate the DVQ-R in the Ecuadorian context, a significant contribution given the paucity of research on dating violence in this population. The validation of this instrument allows for a more accurate assessment of the prevalence of dating violence in university students, which is essential for developing effective prevention policies and intervention programmes in Ecuador (Cherrez Santos et al., 2022). The high internal consistency and construct validity demonstrated in this research further support the applicability of the scale in this context, laying the groundwork for future studies in the region.

### Limitations

This study has certain limitations that should be highlighted. Although the sample size was sufficient to achieve the objectives set and therefore the internal validity of the study can be considered adequate, the fact that it focused only on a population of university students limits its external validity. To overcome this limitation, this design would need to be replicated with more diverse samples of Ecuadorian youth from different educational contexts. Furthermore, although measures were taken to guarantee the maximum anonymity of the people participating in the study and that the nature of their participation was voluntary, the introduction of social desirability bias in the responses to the questionnaire cannot be ruled out. It should also be borne in mind that cultural differences within the university population and the particularities of the academic environment may have played a significant role in shaping the factor structure. Therefore, the importance of considering cultural diversity, even within the same country, and the need to adapt measurement tools to specific contexts, such as the university environment, should be emphasized. Despite these limitations, we believe that the study offers valuable insights into dating violence in the university context in Ecuador. The incorporation of the opinions of the group of experts brought rigour and coherence to the process of cultural adaptation and validation, helping to maintain a close link between the meaning of the items and the constructs to be explored. Finally, the method followed allowed us to design a questionnaire with solid psychometric properties that we hope will prove useful in the future.

## 5. Conclusions

The adaptation of the Dating Violence Questionnaire (DVQ-R) to this sample of Ecuadorian university students highlighted significant differences with respect to the original, both in the structure of the questionnaire and in the characteristics of the sample. The new emerging dimensions, the inclusion of people of both sexes, and the influence of the cultural and university context, as well as the specific attention given to the age variable, offer valuable perspectives for future research. Additionally, these results suggest that the adapted questionnaire could be useful for research in other sociocultural contexts in Latin America, given the similarities in cultural and social patterns across the region. These findings underscore the importance of tailoring measurement tools to the specific dynamics of different populations, which will be essential for developing effective prevention strategies and interventions aimed at addressing IPV in university settings.

## Figures and Tables

**Table 1:** Socio-demographic and time-related variables of relationships in the study population (n = 340).

	Total n = 340	
	M	D.S
Age	25	4.716
	n	%
Sex		
Female	269	79.1
Male	71	20.9
University		
UCSG	208	61.2
UG	132	38.8
Couple		
No	194	57.1
Yes	146	42.9
Time relationship		
<1 month	3	0.9
1–12 months	20	5.9
1–3 ages	43	12.6
>3 ages	80	23.5

**Table 2:** Exploratory factor analysis (EFA) results.

	Communalities	Factor 1	Factor 2	Factor 3	Factor 4
Item 1	0.560				0.741
Item 2	0.694			0.811	
Item 5	0.587		0.755		
Item 6	0.366	0.602			
Item 9	0.545				0.65
Item 10	0.667			0.719	
Item 13	0.619		0.646		
Item 14	0.629	0.613			
Item 15	0.648		0.797		
Item 20	0.594		0.763		
Item 21	0.581	0.712			
Item 23	0.581				0.739
Item 25	0.796			0.900	
Item 26	0.607	0.736			
Item 30	0.523	0.650			
Item 32	0.509				0.537
Item 38	0.643			0.771	
Item 39	0.556	0.687			
Item 40	0.626	0.719			
Item 41	0.615	0.758			
AVE		0.48	0.47	0.60	0.40

**Table 3:** Internal discriminant validity of the scale.

Factor No. 1	Factor No. 2	AVE1	AVE2	ϕ	ϕ^2^	
F1	F2	0.48	0.47	0.33	0.11	SÍ
F1	F3	0.48	0.60	0.51	0.26	SÍ
F1	F4	0.48	0.40	0.48	0.23	SÍ
F2	F3	0.47	0.60	0.23	0.05	SÍ
F2	F4	0.47	0.40	0.29	0.09	SÍ
F3	F4	0.60	0.40	0.35	0.12	SÍ

F1: Emotional Neglect and Contempt Dimension; F2: Coercion and Control Dimension; F3: Physical Violence and Aggression Dimension; F4: Manipulation and Emotional Testing Dimension; AVE1: Average Variance Extracted factor No. 1; AVE2: Average Variance Extracted factor No. 2; ϕ: interfactorial correlation; ϕ^2^: variance.

**Table 4:** Alfa coefficient (α) for the four factors of DVQ-R.

Factor	Number of Items	α
F1. Emotional Neglect and Contempt Dimension	8	0.872
F2. Physical Violence and Aggression Dimension	4	0.764
F3. Coercion and Control Dimension	4	0.849
F4. Manipulation and Emotional Testing Dimension	4	0.729
Total scale	20	0.839

**Table 5:** Expected fit indices for a structural equation model and indices obtained for the confirmatory factor analysis (CFA).

Adjustment Index	Expected	Original Model	4-Factor Model
χ^2^	>0.05	0.001	0.001
CMIN/DF	<5	3.849	1.052
GFI	0.90–1	0.976	0.993
AGFI	0.90–1	0.961	0.989
RMR	≈0.5	0.097	0.048
RMSEA	<0.05	0.092	0.012
CFI	0.90–1	0.971	0.999
NFI	0.90–1	0.962	0.989
TLI	0.90–1	0.966	0.999

Abbreviations: χ^2^: chi-square; CMIN/DF: discrepancy between chi-square and degrees of freedom; GFI: goodness-of-fit index; AGFI: weighted fit index; RMR: root mean square residual index; RMSEA: root mean square error of approximation; CFI: comparative fit index; NFI: normalized fit index; NNFI or TLI: non-normalized fit index.

**Table 6:** Descriptive statistics of the DVQ-R.

	Mean	SD
Factor 1. Emotional Neglect and Contempt Dimension	1.91	0.806
Factor 2. Physical Violence and Aggression Dimension	1.23	0.235
Factor 3. Coercion and Control Dimension	1.51	0.403
Factor 4. Manipulation and Emotional Testing Dimension	2.03	0.788

## Data Availability

Data are contained within the article.

## Appendix A. Dating Violence Questionnaire (DVQ-R)

1. He/she tests your love, setting traps to check if you cheat on him/her if you love him/her or if you are faithful to him/her.
2. You feel obliged (0) to have sex in order not to give explanations.
5. He/she has hit you (5).
6. He/she is good at studying, but he/she is late for appointments, doesn’t keep his/her promises and is irresponsible.
9. Talks to you about love affairs that he/she imagines you have.
10. Insists on touching you in ways that you don’t like and don’t want.
13. Has hit, pushed, shoved, or pulled you.
14. Does not recognize any responsibility for the relationship or for what happens to both of you.
15. Criticizes you, underestimates your character and/or humiliates your self-esteem.
20. Has thrown blunt objects at you.
21. Hurt you with an object.
23. Ridiculed the way you express yourself.
25. Stopped you from leaving.
26. You feel forced to perform certain sexual acts.
30. Ignores your feelings.
32. Stops talking to you or disappears for several days without explanation as a way of showing his/her anger.
38. Invades your space (listens to loud music when you are studying, interrupts you when you are alone, etc.), invades your privacy (reads your messages, listens to your phone conversations, etc.).
39. Forces you to undress when you don’t want to.
40. Has ridiculed or insulted your beliefs, religion, or social class.
41. Ridicules or insults you for the ideas you hold.

Titles for each factor:

Factor 1: Emotional Neglect and Contempt
  - Items: 6, 14, 21, 26, 30, 39, 40 and 41.
  - Description: This factor appears to be related to behaviours involving emotional disregard and neglect in the relationship, including ridiculing beliefs and lack of acknowledgement towards each other’s feelings.
Factor 2: Physical Violence and Aggression
  - Items: 5, 13, 15, and 20.
  - Description: This factor appears to be related to behaviours of physical violence and aggression, including hitting, pushing, and shoving, and humiliation towards the partner.
Factor 3: Coercion and Control
  - Items: 2, 10, 25, and 38.
  - Description: This factor encompasses controlling and coercive behaviours, including unwanted sexual pressures and invasion of the partner’s privacy and personal space.
Factor 4: Manipulation and Emotional Testing
  - Items: 1, 9, 23, and 32.
  - Description: This factor involves manipulative behaviours and emotional testing, such as setting traps to assess fidelity and manipulating each other’s emotions through tactics such as prolonged silence.
