# Correction: Muñoz-Aucapiña et al. (2025). Psychometric Validation of the Dating Violence Questionnaire (DVQ-R) in Ecuadorians. *Behavioral Sciences*, *15*(1), 68

**DOI:** 10.3390/bs15040461

**Published:** 2025-04-03

**Authors:** Miriam Jacqueline Muñoz-Aucapiña, Rosa Elvira Muñoz-Aucapiña, Inmaculada García-García, María Adelaida Álvarez-Serrano, Ana María Antolí-Jover, Encarnación Martínez-García

**Affiliations:** 1 Department of Nursing, Faculty of Health Sciences, University Católica Santiago de Guayaquil, Guayaquil 090615, Ecuador; miriam.munoz@cu.ucsg.edu.ec (M.J.M.-A.); rosa.munoz@cu.ucsg.edu.ec (R.E.M.-A.); 2 Department of Nursing, Faculty of Health Sciences, University of Granada, 18016 Granada, Spain; igarcia@ugr.es (I.G.-G.); emartinez@ugr.es (E.M.-G.); 3 Department of Nursing, Faculty of Health Sciences, University of Granada, 51001 Ceuta, Spain; antolijover@ugr.es; 4 Virgen de las Nieves University Hospital, 18014 Granada, Spain; 5 Instituto de Investigación Biosanitaria ibs.GRANADA, Granada, Spain

**Updating Affiliation**

In the original publication (Muñoz-Aucapiña et al., 2025), the affliation has now been approved for updating as Instituto de Investigación Biosanitaria ibs.GRANADA, Granada, Spain.

**Deleting Citation**

In the original publication (Muñoz-Aucapiña et al., 2025), Verbeek et al. (2023) was cited. The citation has now been approved for removal in Materials and Methods, *Instrument*, Paragraph Number 1 and the correct text should read as follows:

The questionnaire was divided into two blocks. The first collected information on socio-demographic variables: year of birth; sex (male, female); university (Universidad Católica de Santiago de Guayaquil, Universidad Estatal de Guayaquil); relationship status (No, Yes); relationship duration (1–3 months, 3–6 months, 1–3 years, >3 years). The second block was based on the Dating Violence Questionnaire—Revised (DVQ-R) (Rodríguez Díaz et al., 2017), which was later validated for samples of Ecuadorian women aged 18 to 30 by Cherrez Santos et al. (2022). This study remains the ideal reference for exclusively female samples in this age group and population. This instrument is a simplified version of the Dating Violence Questionnaire (DVQ) (Rodríguez Franco et al., 2010). It is aimed at adolescents involved in dating relationships, or who have been in a relationship in the last six months, with a minimum duration of one month. It includes 20 items and uses a Likert-type scale with five response options (0 = Never to 4 = Always). It can be administered individually or in a group, lasting approximately five to ten minutes. This questionnaire assesses five dimensions of dating violence, which are shown below:
